# Effects of the *Dating Matters®* Comprehensive Prevention Model on Health- and Delinquency-Related Risk Behaviors in Middle School Youth: a Cluster-Randomized Controlled Trial

**DOI:** 10.1007/s11121-020-01114-6

**Published:** 2020-04-03

**Authors:** Lianne Fuino Estefan, Alana M. Vivolo-Kantor, Phyllis Holditch Niolon, Vi D. Le, Allison J. Tracy, Todd D. Little, Sarah DeGue, Natasha E. Latzman, Andra Tharp, Kyle M. Lang, Wendy LiKamWa McIntosh

**Affiliations:** 1 Division of Violence Prevention, National Center for Injury Prevention and Control, Centers for Disease Control and Prevention, 4770 Buford Highway NE, Mailstop S106-10, Atlanta, GA 30341 USA; 2 2M Research, Arlington, TX USA; 3 Institute for Measurement, Methodology, Analysis and Policy, Texas Tech University, Lubbock, TX USA

**Keywords:** Teen dating violence, Dating matters, Prevention, Substance use, Weapon, Delinquency

## Abstract

Teen dating violence (TDV) is associated with a variety of delinquent behaviors, such as theft, and health- and delinquency-related risk behaviors, including alcohol use, substance abuse, and weapon carrying. These behaviors may co-occur due to shared risk factors. Thus, comprehensive TDV-focused prevention programs may also impact these other risk behaviors. This study examined the effectiveness of CDC’s *Dating Matters®: Strategies to Promote Healthy Teen Relationships (Dating Matters)* comprehensive TDV prevention model compared to a standard-of-care condition on health- and delinquency-related risk behaviors among middle school students. Students (*N* = 3301; 53% female; 50% black, non-Hispanic; and 31% Hispanic) in 46 middle schools in four sites across the USA were surveyed twice yearly in 6th, 7th, and 8th grades. A structural equation modeling framework with multiple imputation to account for missing data was utilized. On average over time, students receiving *Dating Matters* scored 9% lower on a measure of weapon carrying, 9% lower on a measure of alcohol and substance abuse, and 8% lower on a measure of delinquency by the end of middle school than students receiving an evidence-based standard-of-care TDV prevention program. *Dating Matters* demonstrated protective effects for most groups of students through the end of middle school. These results suggest that this comprehensive model is successful at preventing risk behaviors associated with TDV. clinicaltrials.gov Identifier: NCT01672541

## Introduction

Teen dating violence (TDV)—defined by the Centers for Disease Control and Prevention (CDC) as physical, sexual, psychological, or emotional violence, including stalking, within a dating relationship (Centers for Disease Control and Prevention 2017b)—is a significant public health problem. Results from the 2017 national Youth Risk Behavior Surveillance System (YRBSS) indicate that, of high school students who reported dating, approximately 8% were victims of some form of physical TDV and 7% were victims of some form of sexual TDV in the past 12 months (Kann et al. 2018). Rates of TDV perpetration are also high, particularly in disadvantaged communities. Although national data comparing disadvantaged and more advantaged neighborhoods is not available, youth in high-risk environments may be exposed to risk factors that put them at higher risk for experiencing TDV (Foshee et al. 2012). In baseline data from the *Dating Matters* study, a small sample of middle school students from “high-risk” communities, 32% of students who had dated reported perpetrating physical abuse, 15% reported perpetrating sexual abuse, and 77% reported perpetrating verbal/emotional abuse of a dating partner (Niolon et al. 2015).

### Association Between Teen Dating Violence and Risk Behaviors

Research suggests TDV may often occur as part of a broader, interrelated constellation of risk behaviors (Vivolo-Kantor et al. 2016), a collection of actions that includes other forms of interpersonal violence as well as non-violent risk behaviors. TDV is associated with a variety of health- and delinquency-related risk behaviors in adolescence, including substance use (Exner-Cortens et al. 2013; Silverman et al. 2001), weapon carrying Vagi et al. 2015; Vivolo-Kantor et al. 2016), and delinquent behaviors such as truancy, stealing, and damaging property (Exner-Cortens et al. 2013). Substance use has been consistently linked to TDV exposure in cross-sectional studies (Johnson et al. 2017; Silverman et al. 2001; Vagi et al. 2015). For example, findings from the 2013 national YRBS indicate that high school students who experienced any physical or sexual TDV victimization in the past year were more likely to report current alcohol use, binge drinking, current marijuana use, and ever having used cocaine than students who had not experienced TDV (Vagi et al. 2015). Further, although few longitudinal studies exist on the consequences of TDV on future substance use, there is evidence that TDV victimization may predict subsequent substance use and abuse (Exner-Cortens et al. 2013). Finally, recent studies suggest that there may be a bidirectional relationship between substance use and TDV victimization and perpetration. Taylor and Sullivan (2017) found that substance use may increase risk for physical and psychological TDV victimization among early adolescents in addition to potentially serving as a coping mechanism for victims. Another longitudinal study found that TDV perpetration significantly predicted later marijuana use (Foshee et al. 2016), and multiple longitudinal studies have demonstrated that substance abuse is a risk factor for engagement in TDV perpetration (Foshee et al. 2015; Rothman et al. 2012; Vagi et al. 2013).

Weapon carrying (e.g., guns, knives, and clubs) has also been associated with TDV in several studies. Data from the 2013 YRBS demonstrated that high school students who experienced physical or sexual TDV were more likely than other youth to report carrying a weapon on one or more days in the past 30 days (Vagi et al. 2015; Vivolo-Kantor et al. 2016). Although both male and female TDV victims were threatened or injured with a weapon on school property more often than non-victims, male victims had significantly higher mean scores than females, suggesting males may be at greater risk for experiencing other forms of violence in school (Vivolo-Kantor et al. 2016).

Other delinquency-related risk behaviors, such as property destruction, theft, and runaway behaviors, are also associated with TDV. In two studies from the National Longitudinal Study of Adolescent Health, TDV victimization was associated with delinquency-related behaviors 1 year (Roberts et al. 2003) and 5 years (Exner-Cortens et al. 2013) after victimization. Further, Exner-Cortens et al. (2013) found that the persistence of delinquency-related behaviors over time was most pronounced for males.

### Prevention Programming That Addresses Violence and Associated Risk Factors

Researchers have called for cross-cutting prevention strategies—programs that address TDV, other forms of violence, and associated risk behaviors that may not involve interpersonal violence (e.g., alcohol and substance abuse, delinquency)—to address shared risk factors (Centers for Disease Control and Prevention 2016; DeGue et al. 2013; Vivolo et al. 2010). Given the overlap of violence and other risk behaviors, it is possible that TDV-focused prevention programs will also have impacts on health- and delinquency-related risk behaviors. One way to examine this is to study whether existing evidence-based programs have effects on related risk and protective factors (DeGue et al. 2013). Indeed, efforts to explore cross-over effects for prevention interventions are becoming increasingly common (Reider and Sims 2016). To date, however, only a few evaluations of TDV prevention programs have examined cross-over effects and with mixed results. For example, a study of *Safe Dates* found that students who participated in this effective TDV prevention program were 31% less likely to carry a weapon than students in the control condition at 1-year follow up (Foshee et al. 2014). In a cluster-randomized trial of *Fourth R*, where outcomes were assessed at 2.5 years post intervention, no association was found between exposure to the intervention and substance use (Wolfe et al. 2009). However, other studies of *Fourth R* have found a relationship between the intervention and violent delinquency in high-risk subsamples (Crooks et al. 2011; Crooks et al. 2007).

To continue to move the field forward, CDC developed a comprehensive TDV prevention model, *Dating Matters®: Strategies to Promote Healthy Teen Relationships (Dating Matters)*. *Dating Matters* was developed between 2009 and 2011 and designed for middle school youth aged 11 through 14 years old (6th through 8th grade) with the goal of promoting healthy relationships and preventing TDV. *Dating Matters*’ comprehensive approach goes beyond single-component TDV prevention programs by employing a variety of strategies at multiple levels of the social ecology to accomplish these goals, including strategies for youth, parents, educators, and the community (CDC 2017a; Teten Tharp 2012; Teten Tharp et al. 2011). Results from a cluster-randomized controlled trial of the *Dating Matters* comprehensive prevention model found that by the end of 8th grade, students who participated in *Dating Matters* had lower levels of TDV perpetration and victimization and use of negative conflict resolution strategies compared to students in the standard-of-care condition, Foshee et al.’s (2014) evidence-based TDV prevention program *Safe Dates* (Niolon et al. 2019). In addition, students who participated in *Dating Matters* reported lower levels of bullying perpetration, cyberbullying perpetration and victimization, and physical violence perpetration (Vivolo-Kantor et al. under review) compared to students in the standard-of-care condition.

Given the possible clustering of risk behaviors among some adolescents, *Dating Matters* may also have positive effects beyond these violence outcomes and may prevent or reduce delinquent and risk behaviors among youth. The overall framing of the *Dating Matters* youth programs is on healthy relationships, and the youth programs include material dedicated to emotion management, emotional literacy, and making healthy, safe decisions—all of which may contribute to the reduction of multiple risky behaviors. In addition, some sessions within the youth programs address risky behaviors in the context of other discussions. For example, one session utilizes alcohol and substance abuse as an example of problematic coping behavior, and another session discusses how these substances may be used by perpetrators to coerce unwanted sexual behavior. Finally, *Dating Matters* is a comprehensive intervention that targets multiple levels of the social ecology—the individual, relationship, and community levels—which may help to reduce multiple forms of risky behavior in youth (Teten Tharp et al. 2011). Other components of *Dating Matters*, including the parent programs and i2i youth communications program, likely impact these outcomes through targeting positive parenting skills, parent-child communication, and parental supervision as well as reinforcing the overall messaging from the youth programs.

Identifying cross-over effects on risk behaviors beyond violence maximizes resources invested in the development and evaluation of that intervention and may save time and resources that might otherwise be expended to develop, implement, and evaluate a new intervention. As schools and communities experience greater prevention needs and requirements in the context of limited prevention resources, interventions that have effects on multiple problem behaviors are highly desirable and represent an efficient use of resources.

### The Present Study

The purpose of the current study is to assess the effects of the *Dating Matters* (DM) comprehensive TDV prevention model, compared to a standard-of-care (SC) program, on health- and delinquency-related risk behaviors (i.e., behaviors that are not explicitly interpersonal violence) among middle school students. Specifically, we hypothesized that students exposed to DM will report less weapon carrying, alcohol and substance use, and delinquent behaviors compared to students in the SC condition.

## Method

This study draws from a larger multi-site, cluster-randomized controlled trial to evaluate intervention effects on teens’ dating behaviors, peer relationships, and other outcomes. Details on the methods are available in a previous publication from these data (Niolon et al. 2019). Here we provide a brief summary of the methods employed. All procedures and materials were approved by multiple Institutional Review Boards and the Office of Management and Budget (OMB #0920–0941).

### Design

We randomly assigned middle schools serving high-risk communities in four cities to receive the *Dating Matters* comprehensive prevention model (DM, *N* = 22) during 6th–8th grades, or the evidence-based standard-of-care program, *Safe Dates*, a classroom-based program delivered to 8th grade students only (SC, *N* = 24). In this study, high-risk communities were defined as those that had above average crime and above average economic disadvantage in comparison to the rest of the city or the state. The study design allowed us to test the effects of *Dating Matters* over and above an already evidence-based TDV prevention program. This meant that all students were receiving an intervention, an important consideration in the high-risk communities in which the study was conducted. We replaced schools that dropped out in the first 3 years of the study (*n* = 12) with demographically similar schools on a rolling basis, randomly assigning each to a condition. The two prevention strategies (DM and SC) were implemented during four consecutive school years, starting in the 2012–2013 school year. Students in 6th, 7th, and 8th grade received a survey in fall and spring of each school year (fall 2012–spring 2016), totaling six surveys. We added new cohorts of 6th graders in 2013–2014 and 2014–2015 for a total of five cohorts. Parental permission was obtained prior to approaching students to take a survey, and informed assent was obtained from all participants prior to surveying (Niolon et al. 2016). The overall survey participation rate was 79.7%. Copies of the surveys are available upon request.

### Sample

We analyzed data from students who attended a school that had implemented either program for at least two full academic years (DM: *N* = 22; SC: *N* = 24; see Niolon et al. 2019 for more information). Schools were omitted from the analysis if they did not contribute data or did not participate for at least two full academic years (DM: *N* = 8; SC: *N* = 4). Omitted schools tended to have a lower student-teacher ratio and a smaller student body than those retained, although racial/ethnic composition did not differ substantially. The decision to include schools for analysis based on two full years of participation was based on several reasons. These reasons included the fact that schools implementing less than 2 years would have implemented less than half of the 3-year middle school span covered by the DM components, and students from those schools would have less than half of the opportunities to participate in survey data collection.

We also limited analyses to two cohorts (cohorts 3 and 4) who entered the study in 6th grade and completed 8th grade by the end of the study (total sample: 3301; DM: *N* = 1662, SC: *N* = 1639). These two “full-exposure” cohorts had the opportunity to receive all 3 years of intervention components in the DM condition. The sample consisted of 1750 females (53%) and 1551 males (47%), and the mean age was 11.93 years in the fall semester of 6th grade (SD = 0.57). Students were predominantly black, non-Hispanic (*N* = 1641, 50%) and Hispanic (*N* = 1022, 31%). Fewer students were white, non-Hispanic (*N* = 136, 4%); Asian, non-Hispanic (*N* = 233, 7%); non-Hispanic multiracial (*N* = 232, 7%); and Native American/Alaskan native or Native Hawaiian/other Pacific Islander (less than 1%). We found small but significant baseline differences in racial ethnic composition (max Cox = 0.39); there were more Hispanic and fewer white and black students in schools assigned to the SC condition. There were no baseline differences with regard to age (max Hedge’s *g* = 0.01).

### Prevention Model

The *Dating Matters* comprehensive prevention model (Niolon et al. 2019; Teten Tharp 2012; Teten Tharp et al. 2011) consists of multiple complementary components: (1) classroom-delivered programs for youth in 6th, 7th, and 8th grades; (2) community-based parent programs for parents of 6th, 7th, and 8th grade youth; (3) a school-level intervention (educator training for all educators in schools receiving DM); (4) a “near-peer”-led youth communications program; and (5) community-level activities to promote capacity and readiness assessment, policy development, and use of indicator data. This prevention model addresses both the prevention of TDV and multiple risk factors for TDV, including those related to delinquent behaviors. Additional information on the prevention model can be found in Niolon et al. (2019) and online at www.cdc.gov/violenceprevention/datingmatters.

### Measures

#### Weapon Carrying

Weapon carrying in the past 30 days was assessed with a single item from the YRBS, rated on a 1-to-5 scale: “During the past 30 days, on how many days did you carry a weapon such as a gun, knife, or club?” Response options were 0 day, 1 day, 2 or 3 days, 4 or 5 days, and 6 or more days (Kann et al. 2016).

#### Alcohol and Other Drug Use

Adolescents’ use of alcohol and other drugs was measured by the Adolescent Substance Involvement measure (Knight et al. 2008). This measure assessed the frequency in the past year of the following behaviors: (1) drank more than a sip or taste of beer, wine, wine coolers, or liquor (like whiskey or gin); (2) smoked cigarettes; (3) been drunk; (4) used marijuana or weed (pot, hash, reefer); (5) used inhalants (sniffing glue, huffing, whippets); (6) used other illegal drugs (cocaine, crack, meth, heroin); (7) used a prescription drug when it was not prescribed for you or that you took only for the experience or feeling it caused. Responses to these items, rated on a 1-to-5 scale, were never, 1 or 2 times, 3 to 5 times, 6 to 9 times, and 10 or more times. Average Cronbach’s alpha across time and analysis groups was 0.78.

#### Delinquent Behaviors

Questions assessing adolescents’ involvement in delinquency drew from the National Longitudinal Study of Adolescent Health (Udry 2003). The following six items were rated on a 1-to-4 scale: “In the past 6 months [baseline survey version]/4 months [follow-up survey version], how often did you: (1) deliberately damage property that didn’t belong to you (including painting graffiti or signs); (2) get into a serious physical fight; (3) run away from home; (4) steal something worth more than $50; (5) sell marijuana or other drugs; (6) steal something worth less than $50?” Response options were never, 1 or 2 times, 3 or 4 times, and 5 or more times. Average Cronbach’s alpha across time and analysis groups was 0.70 (Harris et al. 2009).

### Modeling Approach

Data for students in cohorts 3 and 4 were imputed if the student participated in at least one survey. Multiple imputation of missing data (Lang et al. 2017) was conducted under the assumption of missing at random for the demographics and outcome variables. School dropout and replacement resulted in an average of 7% missing data in the student-level outcome scores. Within participating schools, entry and exit of students (e.g., transfer students, opt out) resulted in an average of 43% missing data across all waves. Among students who took the survey, item non-response accounted for an average of 17% for delinquency items, 16% of weapon carrying items, and 12% for alcohol and other drug use items. Next, we conducted several pre-analysis data preparation steps. Outcomes were first scaled to a “percent of maximum score” metric (POMS), which ranges from 0 to 100. For example, if a student responded “never” to all 7 substance use items, a POMS score of 0 was assigned. If the student responded “10 or more times” to all items, a score of 100 was assigned. Once rescaled, we adjusted the outcomes with respect to covariates. To adjust for the non-independence of observations produced in a clustered sample (i.e., students nested within schools), we included a set of indicators of school membership in a pre-analysis covariate adjustment model. We also included race/ethnicity, age, survey date at each time point, guardianship status, and witnessing violence. Outliers in the distributions of the residualized outcome scores were corrected. Details of the covariate adjustment and outlier correction models are provided in the supplemental material in Niolon et al. (2019).

### Statistical Analysis

We used an eight-group structural equation modeling framework (treatment condition by sex and cohort) to assess the equivalence of outcomes at six time points using Mplus V7.4 (Muthen and Muthen 2012). Rather than conducting separate statistical tests of treatment effects for all groups and time points, we used a process designed to impose parsimony on the model parameters (i.e., latent variable means; see Little and Lopez 1997 for an example of this approach). To construct the models, we first freely estimated these 48 means and then iteratively applied equality constraints to identify statistically similar means. Formal tests of these constraints were evaluated using a chi-square difference test between the unconstrained and constrained models, using a stringent criterion to evaluate overall model fit (*p* > 0.2). Models were also evaluated for local fit. Means that were not significantly different were constrained to be equal; therefore, any differences in means depicted in the figures represent statistically significant differences between groups. While this method of hypothesis testing is not common, it is well-suited to our study because independent hypothesis tests of many program effects across groups and time points inflate the potential for “false positives.” Applying a correction factor to the significance tests to control this error would result in an increased chance of overlooking promising evidence of program effects (“false negatives”). Our chosen modeling approach balances the risk of these types of error.

## Results

Baseline equivalence was supported for all outcomes. The magnitude of prevention effects is estimated as the difference between DM and SC students and presented in two ways: (1) group mean values in POMS scale at each time point and (2) reduction in relative risk (RR) for DM students relative to their SC counterparts within the same sex/cohort group.

### Weapon Carrying

**Fig. 1:**
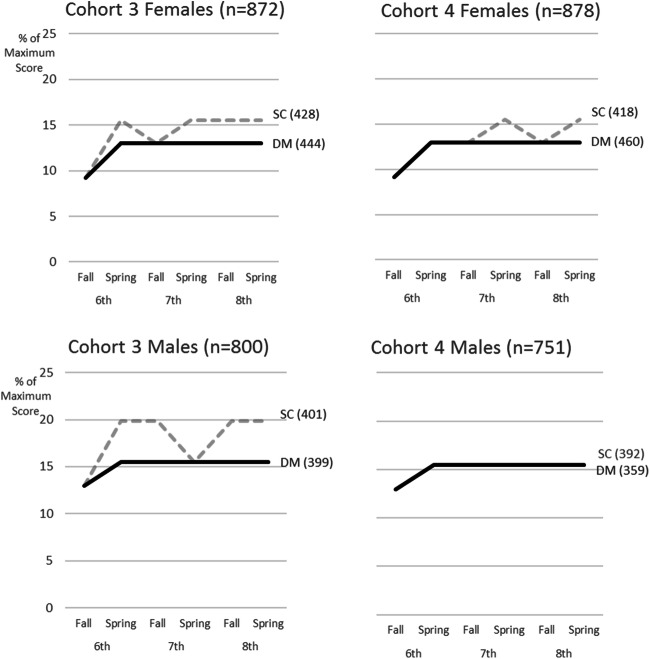
Weapon carrying across time by sex and cohort. SC standard-of-care condition, DM Dating Matters condition. Percent of maximum score (POMS) refers to the maximum possible score given the number of items and response categories in a scale, rather than the maximum observed score. Mean POMS scores have been constrained to appear equal when not significantly different; non-overlapping lines at any time point represent a statistically significant group difference

Four mean constraints described all 48 means without significantly degrading the fit of the freely estimated model (see Table 1). Significant protective intervention effects were found for all groups except cohort 4 males (see Fig. 1). Differences in weapon carrying between DM and SC students averaged 1.62 POMS (range = 0.00–4.35). The average relative risk reduction was 9% (range = 0–22%) (see Fig. 4). For both cohorts of females and for cohort 3 males, DM students had significantly lower weapon carrying scores than SC students by spring of 8th grade, with relative risk reductions between DM and SC at this time point ranging from 16 to 22%.

**Table 1:** Model results and model-estimated means: weapon carrying

Model results: weapon carrying										
Unconstrained				Constrained				Difference		
Chi-square	*df*	RMSEA	SRMR	Chi-square	*df*	RMSEA	SRMR	Chi-square	*df*	*p* value
0.00	0	0.00	0.00	32.68	44	0.00	0.03	32.68	44	0.896
		Rank	Mean			Wald	*p* value			
		1	9.18	1 v 2		− 3.21	0.001			
		2	12.98	2 v 3		− 3.57	0.000			
		3	15.49	3 v 4		− 3.17	0.002			
		4	19.84							
Model-estimated means: weapon carrying										
				*N*	Fall 6th	Spring 6th	Fall 7th	Spring 7th	Fall 8th	Spring 8th
SC females—cohort 3				428	9.18	15.49	12.98	15.49	15.49	15.49
SC males—cohort 3				401	12.98	19.84	19.84	15.49	19.84	19.84
DM females—cohort 3				444	9.18	12.98	12.98	12.98	12.98	12.98
DM males—cohort 3				399	12.98	15.49	15.49	15.49	15.49	15.49
SC females—cohort 4				418	9.18	12.98	12.98	15.49	12.98	15.49
SC males—cohort 4				392	12.98	15.49	15.49	15.49	15.49	15.49
DM females—cohort 4				460	9.18	12.98	12.98	12.98	12.98	12.98
DM males—cohort 4				359	12.98	15.49	15.49	15.49	15.49	15.49

*SC* standard condition, *DM* Dating Matters Comprehensive condition

### Alcohol and Other Drugs

**Fig. 2:**
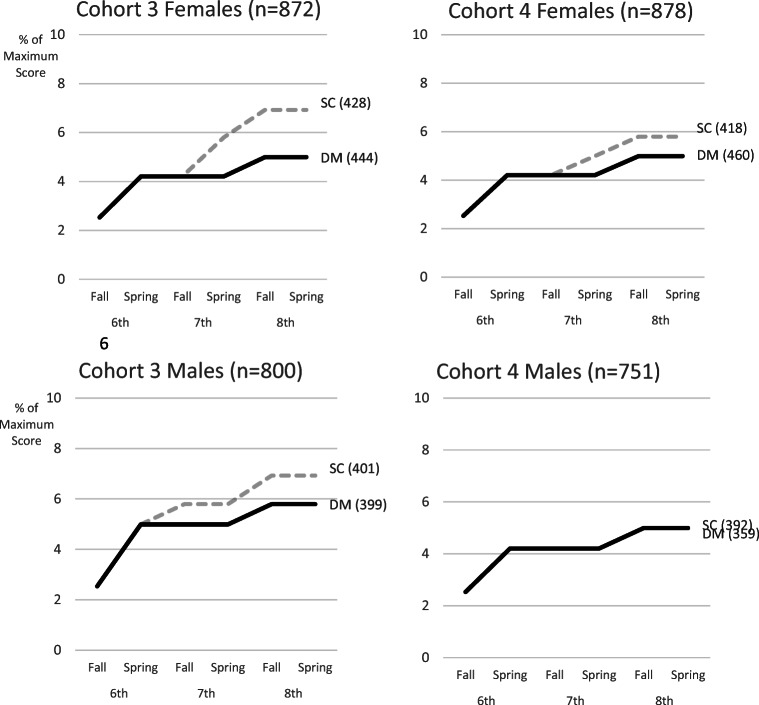
Alcohol and other drugs across time by sex and cohort. SC standard-of-care condition, DM Dating Matters condition. Percent of maximum score (POMS) refers to the maximum possible score given the number of items and response categories in a scale, rather than the maximum observed score. Mean POMS scores have been constrained to appear equal when not significantly different; non-overlapping lines at any time point represent a statistically significant group difference

Five mean constraints described all 48 means without significantly degrading the fit compared with the freely estimated model (see Table 2). Significant intervention effects were found for all groups except males in cohort 4, for which no program effects emerged (see Fig. 2). Differences in alcohol and drug use between DM and SC students averaged 0.59 POMS (range = 0.00–1.93). The average relative risk reduction was 9% (range = 0–28%) (see Fig. 4). For both cohorts of females and for cohort 3 males, DM students had significantly lower alcohol and drug use scores than SC students by spring of 8th grade, with relative risk reductions between DM and SC at this time point ranging from 14 to 28%.

**Table 2:** Model results and model-estimated means: alcohol and other drugs

Model results: alcohol and other drugs										
Unconstrained				Constrained				Difference		
Chi-square	*df*	RMSEA	SRMR	Chi-square	*df*	RMSEA	SRMR	Chi-square	*df*	*p* value
0.00	0	0.00	0.00	49.07	43	0.02	0.03	49.07	43	0.243
		Rank	Mean			Wald	*p* value			
		1	2.53	1 v 2		− 9.70	0.000			
		2	4.20	2 v 3		− 5.11	0.000			
		3	4.99	3 v 4		− 3.61	0.000			
		4	5.80	4 v 5		− 3.40	0.001			
		5	6.93							
Model-estimated means: alcohol and other drugs										
				*N*	Fall 6th	Spring 6th	Fall 7th	Spring 7th	Fall 8th	Spring 8th
SC females—cohort 3				428	2.53	4.20	4.20	5.80	6.93	6.93
SC males—cohort 3				401	2.53	4.99	5.80	5.80	6.93	6.93
DM females—cohort 3				444	2.53	4.20	4.20	4.20	4.99	4.99
DM males—cohort 3				399	2.53	4.99	4.99	4.99	5.80	5.80
SC females—cohort 4				418	2.53	4.20	4.20	4.99	5.80	5.80
SC males—cohort 4				392	2.53	4.20	4.20	4.20	4.99	4.99
DM females—cohort 4				460	2.53	4.20	4.20	4.20	4.99	4.99
DM males—cohort 4				359	2.53	4.20	4.20	4.20	4.99	4.99

*SC* standard condition, *DM* Dating Matters Comprehensive condition

### Delinquency

**Fig. 3:**
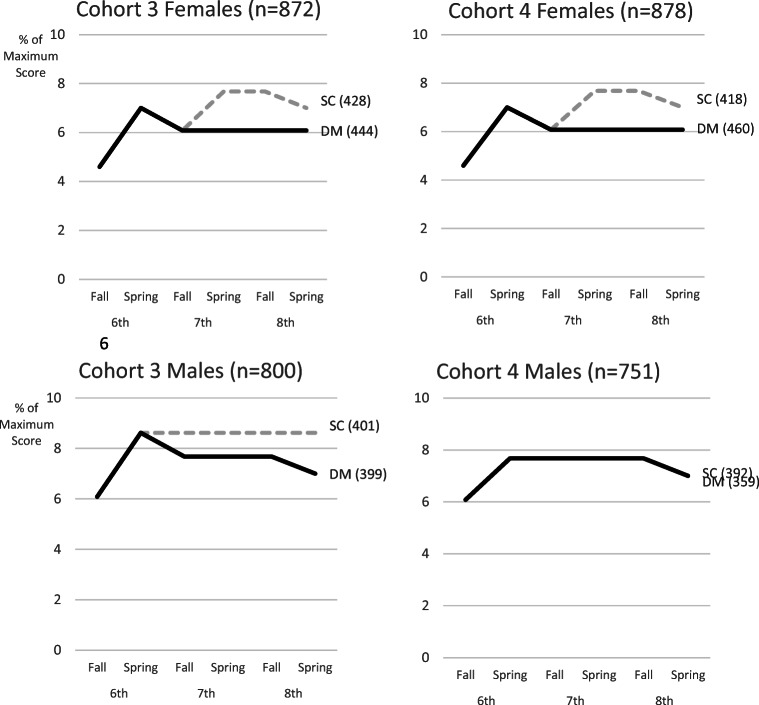
Delinquency across time by sex and cohort. SC standardof-care condition, DM Dating Matters condition. Delinquency was measured using items from the National Longitudinal Study of Adolescent Health. Percent of maximum score (POMS) refers to the maximum possible score given the number of items and response categories in a scale, rather than the maximum observed score. Mean POMS scores have been constrained to appear equal when not significantly different; non-overlapping lines at any time point represent a statistically significant group difference

**Fig. 4:**
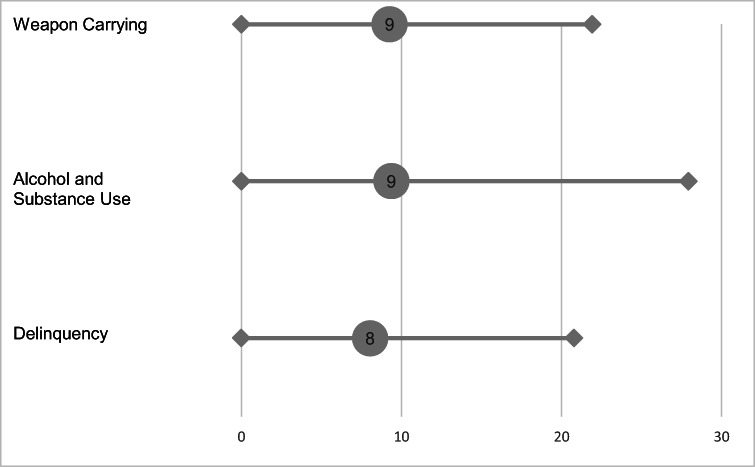
Percent relative risk reduction by outcome (*M*, range) for Dating Matters vs. standard-of-care. Relative risk reduction represents the percent reduction in scores on measures of weapon carrying, alcohol and substance abuse, and delinquency for the Dating Matters Comprehensive condition relative to the standard-of-care condition. The numbers within the circles represent the average risk reduction for that outcome across the 4 groups (cohort × sex), and the space between the diamonds represent the range of relative risk reduction on that outcome across the four groups

Four mean constraints described all 48 means without significantly degrading the fit compared with of the freely estimated model (see Table 3). Significant intervention effects were found for all groups except males in cohort 4, for which no program effects emerged (see Fig. 3). Differences in delinquent behaviors between DM and SC students averaged 0.63 POMS (range = 0.00–1.62). The average relative risk reduction was 8% (range 0–21%) (see Fig. 4). For both cohorts of females and for cohort 3 males, DM students had significantly lower delinquent behavior scores than SC students by spring of 8th grade, with relative risk reductions between DM and SC at this time point ranging from 13 to 19%.

**Table 3:** Model results and model-estimated means: delinquency

Model results: delinquency										
Unconstrained				Constrained				Difference		
Chi-square	*df*	RMSEA	SRMR	Chi-square	*df*	RMSEA	SRMR	Chi-square	*df*	*p* value
0.00	0	0.00	0.00	26.70	43	0.00	0.02	26.70	43	0.976
		Rank	Mean			Wald	*p* value			
		1	4.59	1 v 2		− 4.99	0.000			
		2	6.08	2 v 3		− 4.15	0.000			
		3	7.00	3 v 4		− 3.01	0.003			
		4	7.68							
Model-estimated means: delinquency										
				*N*	Fall 6th	Spring 6th	Fall 7th	Spring 7th	Fall 8th	Spring 8th
SC females—cohort 3				428	4.59	7.00	6.08	7.68	7.68	7.00
SC males—cohort 3				401	6.08	8.62	8.62	8.62	8.62	8.62
DM females—cohort 3				444	4.59	7.00	6.08	6.08	6.08	6.08
DM males—cohort 3				399	6.08	8.62	7.68	7.68	7.68	7.00
SC females—cohort 4				418	4.59	7.00	6.08	7.68	7.68	7.00
SC males—cohort 4				392	6.08	7.68	7.68	7.68	7.68	7.00
DM females—cohort 4				460	4.59	7.00	6.08	6.08	6.08	6.08
DM males—cohort 4				359	6.08	7.68	7.68	7.68	7.68	7.00

*SC* standard condition, *DM* Dating Matters Comprehensive condition

## Discussion

This paper examines effects of the *Dating Matters* comprehensive TDV prevention model compared to a standard-of-care condition on delinquency behaviors among middle school students. Overall, results supported the hypothesis that *Dating Matters* reduces students’ risk for weapon carrying, delinquent behaviors, and alcohol and other drug use relative to the standard-of-care TDV prevention program, *Safe Dates.* While effects varied across cohort and sex, the overall pattern of findings suggests protective effects of the *Dating Matters* model.

An exception to this overall pattern was found for cohort 4 males. The findings for cohorts 3 and 4 males in Figs. 1, 2, and 3 suggest very similar patterns of scores for males in the *Dating Matters* condition across cohorts. However, cohort 4 males in the standard-of-care condition do not seem to have experienced the same increase in risk behaviors over time as cohort 3 males in the standard-of-care condition. The reason for this pattern is unclear. While there were slight baseline differences between cohort 3 and 4 males on race and ethnicity, these variables were included in the control variables and thus would not explain the differences. Further analyses of these data, such as examining whether site-level differences or implementation fidelity factors result in differences in risk behavior outcomes, may provide further insight into this pattern.

Significant program effects were found for weapon carrying, and reports of weapon carrying remained relatively stable over time for students in the *Dating Matters* condition. Students attending schools implementing *Dating Matters* scored 9% lower on average on a measure of weapon carrying than students attending schools implementing the standard-of-care. Although scores on weapon carrying varied over time in the standard-of-care condition, levels were generally higher than in the *Dating Matters* condition. This finding is particularly notable considering that in prior trials of *Safe Dates*, which served as our standard-of-care condition, the program was found to reduce reports of weapon carrying by 31% relative to students not receiving an intervention (Foshee et al. 2014). While it is not possible to know whether *Safe Dates* alone had similar effects in the current trial, *Dating Matters* appears to have reduced weapon carrying above and beyond the previously documented effects of *Safe Dates*. The fact that *Dating Matters* reduced weapon carrying among both cohorts of females and one cohort of males, relative to the standard-of-care condition, is encouraging, especially given the general stability of this behavior over time and sex differences in weapon carrying. The additional protective benefit of *Dating Matters* may be due to its two additional years of educational content related to social-emotional learning and healthy behaviors, and its focus on engaging the entire school—all students and educators—leading to shifts in school culture and climate that were not directly assessed in this study.

Significant program effects were also found for substance use outcomes for both cohorts of females and males in cohort 3, despite all groups demonstrating an increase in substance use during middle school. Overall, students attending *Dating Matters* schools scored 9% lower on average on a measure of substance use than students attending standard-of-care schools. The developmental increase in substance abuse behaviors that we found in this sample occurring over time in middle school is consistent with findings from studies of alcohol use (Patrick and Schulenberg 2014) and other substance use, including marijuana (Miech et al. 2016) measured in late middle school through the end of high school. However, it is clear that *Dating Matters* has a protective effect on this developmental pattern above and beyond any effects of the *Safe Dates* program alone. This is noteworthy in that other evidence-based TDV prevention programs that have examined substance abuse outcomes, including *Fourth R*, have not found significant program effects on alcohol or substance use (Wolfe et al. 2009). Given the developmental patterns in alcohol and substance use, this finding highlights the importance of implementing comprehensive prevention programs as early as possible—before risky behaviors begin.

Significant program effects on delinquency outcomes were also found for both cohorts of females and for males in cohort 3. Overall, students attending *Dating Matters* schools had an average of 8% relative risk reduction in delinquent behaviors as compared to students attending standard-of-care schools. It is encouraging that *Dating Matters* demonstrates promise in reducing delinquency as well as related behaviors, such as dating violence, in the high-risk, urban neighborhoods where the trial was conducted (Niolon et al. 2015). As delinquency-related risk behaviors associated with TDV tend to occur in a constellation, programs intended to prevent one of these behaviors have the potential to reduce other risky behaviors that are likely to co-occur.

Taken together, these findings demonstrate that a comprehensive approach to preventing TDV and promoting healthy relationship behaviors, such as *Dating Matters*, can also be effective at reducing related risk behaviors, such as weapon carrying, delinquency, and substance use. This study adds to the limited literature on the cross-over effects of TDV prevention programs. In addition, it is the first such study for a comprehensive prevention model. Specifically, these findings further reinforce the benefits of implementing comprehensive prevention models to address shared risk and protective factors for different forms of violence, including TDV (Centers for Disease Control and Prevention 2016). Of particular importance are comprehensive strategies that are focused on cross-cutting primary prevention and begin as early as possible. Such approaches move away from “siloed” programs that target one behavior at a time. While *Dating Matters* does not specifically focus on reducing delinquency-related risk behaviors, it does focus on healthy coping behaviors and strategies for making healthy, safe decisions that can apply not only to TDV but to other related behaviors. Because of the many correlations among delinquency-related risk behaviors and between delinquent behaviors and TDV, reducing one of these behaviors has the potential to reduce others, both concurrently and in the future. This efficiency may provide a practical benefit to communities interested in implementing programs to prevent and reduce risky adolescent behavior, especially when there are limited resources to do so.

This study has several limitations. First, the study was conducted in four high-risk, urban communities defined by above average rates of crime and poverty. Conducting the study in these communities led to several challenges, including implementation and evaluation differences between sites; school and participant retention; and differences in school and community contexts (see Niolon et al. 2016, for additional details). In addition, the current intent-to-treat analyses are conservative and do not account for the differences in fidelity or overall student exposure to the intervention that may have occurred between sites. Further, like other studies on violence and risk behaviors, we relied on self-reports of behaviors rather than observations or other sources of information on these risky behaviors. Finally, because we conducted this study in high-risk, urban areas, we do not know if the results will generalize to other types of communities.

Despite these limitations, this study has multiple important strengths. In particular, it is the first and largest multi-site, cluster-randomized controlled trial of a comprehensive TDV prevention model, to date, lending power to the analysis and results. While conducting the study in high-risk, urban communities led to some challenges, the study design provided an opportunity for a rigorous evaluation of *Dating Matters* in multiple locations around the USA. The findings add to our understanding of effective prevention strategies in this high-risk context. In addition, it was a comparative effectiveness trial, in which we compared the comprehensive model to an evidence-based program, providing important information about the added value of a comprehensive prevention approach.

Results from this study are promising, particularly because as cross-over effects from the *Dating Matters* comprehensive prevention model, and given the fact that the standard-of-care condition, an evidence-based TDV prevention program, also demonstrated effects on at least one of the examined outcomes in past research. Future planned analyses will examine these same behaviors in high school to see if the results are sustained over time. Additional research using these data could further explore the longitudinal relationship between TDV and delinquency-related risk behaviors by sex, as well as contextual factors that may impact these outcomes. Future studies should also investigate the effectiveness of *Dating Matters* in other types of communities.

The primary goal of *Dating Matters* is to prevent TDV and promote healthy relationship behaviors, and the comprehensive model has, in fact, been effective in preventing TDV perpetration and victimization and the use of negative conflict resolution behaviors through middle school (Niolon et al. 2019). This study demonstrates that this comprehensive model also appears to have protective effects for most groups of students on delinquency-related risk behaviors through the end of 8th grade, underscoring the benefits of early-onset, comprehensive prevention programming for youth.
